# Anti-tumour effects and pharmacokinetic profile of 17-(5′ -isoxazolyl)androsta-4,16-dien-3-one (L-39) in mice: an inhibitor of androgen synthesis

**DOI:** 10.1054/bjoc.2000.1136

**Published:** 2000-06-02

**Authors:** I P Nnane, B J Long, Y-Z Ling, D N Grigoryev, A M Brodie

**Affiliations:** Department of Pharmacology and Experimental Therapeutics, University of Maryland School of Medicine, Baltimore, MD, 21201, USA

**Keywords:** 17α-hydroxylase/C _17,20_-lyase, 5α-reductase, androgens, prostate cancer, pharmacokinetics

## Abstract

17-(5′-Isoxazolyl)androsta-4,16-dien-3-one (L-39), a novel androstene derivative, was synthesized and evaluated in vitro and in vivo. L-39 showed potent and non-competitive inhibition of human testicular microsomal 17α-hydroxylase/C _17,20_-lyase with an IC _50_ value of 59 n M and *K*_i_ of 22 n M. L-39 also showed potent and competitive inhibition of 5α-reductase in human prostatic microsomes with IC _50_ and *K*_i_ values of 33 and 28 n M respectively. L-39 (5 μM) has also been shown to manifest anti-androgenic activity in cultures of human prostate cancer cell lines (LNCaP) by preventing the labelled synthetic androgen R1881 (5 n M) from binding to the androgen receptors. Androgen-ependent human próstate cancer xenografts (PC-82) were grown in nude mice and the effects of L-39 (50 mg kg^–1^day^–1^) on tumour growth and prostate-specific antigen (PSA) levels were determined after 28 days. L-39 significantly (*P*< 0.01) diminished tumour growth and wet weights to a similar extent as castration or flutamide treatment. L-39 also significantly (*P*< 0.01) reduced serum PSA levels by more than 80% in the mice bearing human prostate cancer xenografts. Pharmacokinetic studies were also conducted in male Balb/c mice. After subcutaneous administration of a single bolus dose, L-39 was rapidly absorbed into the systemic circulation. Peak plasma levels occurred at 0.75 h and then declined with a *t*_1/2_ of 1.51 h. The bioavailability of L-39 after subcutaneous administration was 28.5%. These results demonstrate that L-39 is a potent inhibitor of androgen synthesis and is effective in reducing the growth of human prostate cancer xenografts in nude mice. Although improvements in the bioavailability are necessary, L-39 is a potential lead compound with this profile as an inhibitor of prostate cancer growth. © 2000 Cancer Research Campaign


*British Journal of Cancer* (2000) **83**(1), 74–82
© 2000 Cancer Research Campaign
doi: 10.1054/ bjoc.2000.1136, available online at http://www.idealibrary.com on IDEAL®

### Anti-tumour effects and pharmacokinetic profile of 17-(5′-isoxazolyl)androsta-4,16-dien-3-one (L-39) in mice: an inhibitor of androgen synthesis

**IP Nnane*, BJ Long, Y-Z Ling†, DN Grigoryev and AM Brodie**

Department of Pharmacology and Experimental Therapeutics, University of Maryland School of Medicine, Baltimore, MD 21201, USA

**Summary** 17-(5′-Isoxazolyl)androsta-4,16-dien-3-one (L-39), a novel androstene derivative, was synthesized and evaluated in vitro and in vivo. L-39 showed potent and non-competitive inhibition of human testicular microsomal 17α-hydroxylase/$C_{17,20}$-lyase with an $IC_{50}$ value of 59 nM and $K_i$ of 22 nM. L-39 also showed potent and competitive inhibition of 5α-reductase in human prostatic microsomes with $IC_{50}$ and $K_i$ values of 33 and 28 nM respectively. L-39 (5 μM) has also been shown to manifest anti-androgenic activity in cultures of human prostate cancer cell lines (LNCaP) by preventing the labelled synthetic androgen R1881 (5 nM) from binding to the androgen receptors. Androgen-dependent human próstate cancer xenografts (PC-82) were grown in nude mice and the effects of L-39 (50 mg $kg^{-1}$ $day^{-1}$) on tumour growth and prostate-specific antigen (PSA) levels were determined after 28 days. L-39 significantly ($P < 0.01$) diminished tumour growth and wet weights to a similar extent as castration or flutamide treatment. L-39 also significantly ($P < 0.01$) reduced serum PSA levels by more than 80% in the mice bearing human prostate cancer xenografts. Pharmacokinetic studies were also conducted in male Balb/c mice. After subcutaneous administration of a single bolus dose, L-39 was rapidly absorbed into the systemic circulation. Peak plasma levels occurred at 0.75 h and then declined with a $t_{1/2}$ of 1.51 h. The bioavailability of L-39 after subcutaneous administration was 28.5%. These results demonstrate that L-39 is a potent inhibitor of androgen synthesis and is effective in reducing the growth of human prostate cancer xenografts in nude mice. Although improvements in the bioavailability are necessary, L-39 is a potential lead compound with this profile as an inhibitor of prostate cancer growth. © 2000 Cancer Research Campaign

**Keywords:** 17α-hydroxylase/$C_{17,20}$-lyase; 5α-reductase; androgens; prostate cancer; pharmacokinetics

Prostate cancer is second only to lung cancer as the leading cause of death and is the most prevalent cancer amongst men in the USA and Europe and accounts for about a third of all cancers diagnosed each year in men. In the UK, the incidence of prostate cancer has almost doubled over the last 30 years. Similarly, in 1998, it was estimated that more than 334 500 cases were diagnosed and more than 41 800 men died of the disease in the USA (Dijkman and Debruyne, 1996). The prostate gland is under the influence of androgens and often becomes enlarged in older men. It has clearly been demonstrated that prostate cancers are androgen-dependent and that withdrawal of androgen supply produced favourable response in patients with adenocarcinoma of the prostate (Saunders, 1963; Rosenberg and Eschenbach, 1990). Localized prostate cancer is curable and although the metastatic form of the disease is difficult to manage, endocrine therapy does reduce the death rate from the disease and is first-line treatment for all patients. In the testis and adrenals, 17α-hydroxylase/$C_{17,20}$-lyase converts the $C_{21}$ steroid precursors to the corresponding $C_{19}$ androgens. Testosterone (T) is further converted to the more potent androgen dihydrotestosterone (DHT) by 5α-reductase in the prostate (Bruchovsky and Wilson, 1968). Both T and DHT stimulate prostatic growth, although DHT plays a much more important role than T in the organogenesis and homeostasis of the prostate (Wilson, 1996). Current endocrine therapies such as orchidectomy and luteinizing hormone releasing hormone (LHRH) agonists result in reduced androgen production by the testis and are useful in the early stages of prostate cancer. However, these treatment options fail to alter androgen production by the adrenals that may contribute androgen precursors to the prostate. The addition of flutamide, an anti-androgen to inhibit the action of androgens on the prostate, is only partially effective as the disease frequently progresses due to mutations in the androgen receptor, which can utilize flutamide as an agonist. It would appear that total androgen blockade can be therapeutically more effective than conventional androgen ablation therapy (Labrie et al, 1983). However, more effective therapeutic strategies for combating prostate cancer are needed. This may be achieved through dual inhibition of 17α-hydroxylase/$C_{17,20}$-lyase and 5α-reductase (Li et al, 1992; Klus et al, 1996). Several derivatives of oestrogens, progestins and androgens which diminish androgen levels by inhibiting key enzymes in the androgen synthesis cascade have been described (Ayub and Levell 1987; Angelastro et al, 1989; Nakajin et al, 1989; Jarman et al, 1990; Njar and Brodie, 1999). Currently, ketoconazole, an active imidazole fungicide, is the only 17α-hydroxylase/$C_{17,20}$-lyase inhibitor that is used clinically to reduce testosterone biosynthesis in the treatment of patients with

*Received 9 November 1999*
*Revised 4 February 2000*
*Accepted 10 February 2000*

*Correspondence to:* AM Brodie

*Present address: Department of Pharmaceutical Sciences, Temple University School of Pharmacy, Philadelphia, PA 19140, USA.
†Present address: School of Pharmaceutical Sciences, Beijing Medical University, China.

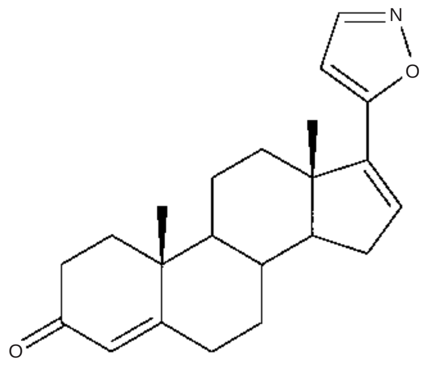

**Figure 1** Structure of 17-(5′-isoxazolyl)androsta-4,16-dien-3-one (L-39)

advanced prostate cancer (Small et al, 1997). However, ketoconazole is not very potent and it inhibits several other steroidogenic enzymes and has a number of significant side-effects (Ayub and Levell, 1987; Trachtenberg, 1984). 5α-Reductase occurs in two isoforms namely, type I and type II. The type II isoenzyme is the predominant form in the human prostate. Finasteride, which was recently approved for the treatment of benign prostatic hypertrophy (BPH), is a more potent inhibitor of the type II than the type I isoform (Cunningham and Hirshkowitz, 1995). However, finasteride is only effective against benign prostatic hypertrophy (BPH) in patients with minimal disease and although the compound reduced DHT levels, it also increased serum T levels (Peters and Sorkin, 1993).

We have previously reported the synthesis and testing of several steroidal inhibitors of 17α-hydroxylase/$C_{17,20}$-lyase and 5α-reductase (Li et al, 1995, 1996; Ling et al, 1997; Njar et al, 1998). These compounds were demonstrated to be effective dual inhibitors of human testicular 17α-hydroxylase/$C_{17,20}$-lyase and prostatic 5α-reductase in vitro (Nnane et al, 1998, 1999; Grigoryev et al, 1999). In animal studies, the compounds diminished the levels of circulating T and DHT in male rat tissues. In LNCaP cell cultures, several of our novel steroidal compounds including 17-(5′-isoxazolyl)androsta-4,16-dien-3-one (L-39; Figure 1) inhibited cellular proliferation and were effective at slowing LNCaP human prostatic cells grown in male severe combined immunodeficient (SCID) mice as tumours. L-39 (5 μM) has also been shown to manifest anti-androgenic activity in cultures of human prostate cancer cell lines (LNCaP) by preventing the labelled synthetic androgen R1881 (5 nM) from binding to the androgen receptors (Long et al, 1999). These compounds, especially L-39, could be more effective than current therapies in the treatment of prostate cancer due to their multiple activities. In the present investigation, we describe the effects of L-39, a novel androstene derivative, in vitro and on human prostate cancer xenografts in nude mice. We also evaluated the pharmacokinetic properties of L-39 in male mice in this investigation.

### MATERIALS AND METHODS

### Chemical inhibitors and reagents

17-(5′-Isoxazolyl)androsta-4,16-dien-3-one (L-39) and its $\Delta^5$ hydroxy derivative (L-38) were synthesized in our laboratory according to procedures described previously (Ling et al, 1997). Finasteride was a gift from Merck Research Laboratories (Rahway, NJ, USA) and ketoconazole was purchased from Sigma Chemical Company (St Louis, MO, USA). [21-$^3$H]-17α-hydroxypregnenolone (13.61 μCi μmol$^{-1}$) was prepared in our laboratory as previously described (Njar et al, 1998). [1,2,6,7-$^3$H]-Testosterone (96.5 Ci mmol$^{-1}$) and [4-$^{14}$C]-dihydrotestosterone (56.5 mCi mmol$^{-1}$) were obtained from Dupont (Boston, MA, USA). Silica gel thin layer chromatographic (TLC) plates (20 × 20 cm, w/uv 254, 500 microns, GF) were obtained from Analtech (Newark, DE, USA). The active [$^{125}$I]-T coated-tube radioimmunoassay (RIA) kits and the [$^{125}$I]-DHT coated tube RIA kits for quantitative measurement of T and DHT, respectively, were purchased from DSL Inc. (Webster, TX, USA). All other reagents were purchased from Sigma Chemical Company (St Louis, MO, USA).

### Preparation of microsomes

Human testes and prostate tissue (from patients with benign prostatic hyperplasia, BPH) were obtained from Dr James Mohler, Director, Urologic Oncology, University of North Carolina at Chapel Hill and stored at –70°C prior to use. Testicular and prostatic microsomes were prepared as described previously (19). Briefly, human testis or prostate was washed with saline (0.9%), blotted dry and weighed. The tissue was minced and homogenized in a blender with two volumes of sucrose (250 mM). The homogenates were added to 50-ml plastic centrifuge tubes and centrifuged at 10 000 ***g*** for 30 min. The resulting supernatant was centrifuged at 109 000 ***g*** for 1 h using an ultra-centrifuge. The microsomal pellet was covered with 2 ml of phosphate buffer (0.1 M) and stored at –70°C until required for assay. The microsomal protein content was determined by the Lowry method (Lowry et al, 1951).

### 17α-hydroxylase/$C_{17,20}$-lyase activity

The measurement of the activity of the human 17α-hydroxylase/$C_{17,20}$-lyase in testicular microsomes, in the absence and presence of inhibitors was performed as described previously (Li et al, 1995). Briefly, the 17α-hydroxylase/$C_{17,20}$-lyase activity was determined by measuring the release of [$^3$H]-acetic acid during the conversion of [21-$^3$H]-17α-hydroxypregnenolone to dehydroepiandrosterone. The incubations were carried out in a total volume of 1.01 ml. Sample tubes were supplied with 10 μl of propylene glycol, 300 000 dpm of [21-$^3$H]-17α-hydroxypregnenolone (13.61 μCi μmol$^{-1}$) and the indicated inhibitors. The control incubations were prepared without the addition of the indicated inhibitors. After evaporation of the ethanolic solution, the following were added to each tube: 750 μl of 0.1 M sodium phosphate buffer (pH 7.4, with 78 μM of dithiothreitol (DTT)) and 50 μl of an NADPH generating system (phosphate buffer containing 6.5 mM of NADP$^+$, 71 mM of glucose-6-phosphate, 1.25 IU of glucose-6-phosphate dehydrogenase). The tubes were pre-incubated for 15 min at 37 °C and the reaction was started by adding 200 μl of human testicular microsomes (300 μg protein per 200 μl of phosphate buffer). The reaction tubes were incubated at 37°C under oxygen. After 1 h, the tubes were placed in an ice bath and steroids in the reaction mixture were extracted two times with chloroform (1 ml). The tubes were allowed to stand at 4°C for 20 min, centrifuged at 4°C for 15 min at 2000 ***g*** and then 0.75 ml of the aqueous phase of each tube was placed into a fresh tube. To remove residual steroids, which may remain after the chloroform extraction, 0.75 ml of charcoal solution (2.5 g of activated charcoal per 100 ml of distilled water) was added to each tube and mixed vigorously. After standing for 30 min, the charcoal was pelleted by centrifugation at 2000 ***g*** for 20 min. Finally, 0.75 ml of the supernatant was analysed for tritium by liquid scintillation spectrometry. The reaction conditions were optimized with 0.1–6.0 µM of [21-$^3$H]-17α-hydroxypregnenolone and the $K_m$ and $V_{max}$ values were determined at the optimum conditions. The optimum conditions were microsomal protein content of 300 µg protein per incubation, a substrate concentration of 0.48 µM, an incubation time of 1 h and a pH of 7.4. The $IC_{50}$ values for inhibitors were calculated using linear regression analysis and the plot of log of enzyme activity against log of inhibitor concentration. The $K_i$ values were also determined at the same reaction conditions with addition of appropriate concentrations of inhibitors. $K_i$ values were calculated from a graph of the slopes of the Lineweaver–Burke plots versus L-39 concentration using the equation of the regression lines. The experiments were performed in duplicate and repeated at least twice (i.e. $n \geq 3$).

### 5α-Reductase assay

The effects of novel compounds on human prostatic 5α-reductase activity were evaluated as previously described (Li et al, 1992) with some modifications. Ethanolic solutions of [1,2,6,7-$^3$H]T (600 000 dpm), cold T (4.8 ng), indicated inhibitors (0–200 nM) and propylene glycol (10 µl) were added to duplicate sample tubes. The control incubations were prepared without the addition of the indicated inhibitors. The ethanol was evaporated to dryness under a gentle stream of air. The samples were reconstituted in phosphate buffer (0.1 M, pH 7.4, 400 µl) containing DTT (78 µM) and the NADPH generating system (NADP, 6.5 mM; glucose-6-phosphate, 71 mM; glucose-6-phosphate dehydrogenase, 2.5 IU, in 100 µl of phosphate buffer) was added to each tube. The tubes were pre-incubated at 37°C for 15 min. The enzymatic reactions were initiated by addition of human BPH microsomes (about 180 µg of microsomal protein in 500 µl of phosphate buffer) in a total volume of 1.01 ml. The incubations were performed for 10 min under oxygen in a shaking water bath at 37°C. The incubations were terminated by placing the sample tubes on ice. [4-$^{14}$C]-DHT (3000 dpm) and cold DHT (50 µg) were added to each tube as an internal standard and visualization marker respectively. These additions were immediately followed by ether (1 ml). The steroids were extracted with ether (3 × 1 ml), separated by TLC (chloroform:ether, 80:20) and visualized by exposure to iodine vapour. The TLC spot corresponding to DHT was scraped, extracted with ether and analysed for $^3$H and $^{14}$C using a liquid scintillation counter. The percentage conversion of [1,2,6,7-$^3$H]T to [1,2,6,7-$^3$H]DHT was calculated and used to determine 5α-reductase activity. The reaction conditions were optimized with T (0–60 nM) and the $K_m$ and $V_{max}$ values were estimated at the optimum conditions. The optimum conditions were microsomal protein content of 180 µg protein per incubation, a substrate concentration of 40 nM, an incubation time of 10 min and a pH of 7.4. The $IC_{50}$ values were determined from log-log plots of 5α-reductase activity against four different concentrations of the inhibitor. The $K_i$ values were also determined at the same reaction conditions with addition of appropriate concentrations of inhibitors. $K_i$ values were calculated from a graph of the slopes from the Lineweaver–Burke plots versus L-39 concentration using the equation of the regression line. The experiments were performed in duplicate and repeated at least twice (i.e. $n \geq 3$).

### Human PC-82 prostate cancer xenograft model

Male athymic Ncr-nu mice (~20 g) obtained from NCI (Frederick, MD, USA) were maintained under sterile conditions in a controlled environment of about 25°C, 50% relative humidity and 12 h of light and 12 h of dark cycles and allowed free access to food and water. The experiments were performed in accordance with guidelines approved by the Veterinary Resources Unit of the University of Maryland School of Medicine, Baltimore. PC-82 tumour was originally kindly provided by Dr John Isaacs (John Hopkins University). The PC-82 tumour (1000 mg) was minced in pieces of 1–2 mm$^3$, suspended in Matrigel (10 ml) and an aliquot (0.1 ml) transplanted with a Trocar needle (18 gauge) into both right and left flanks of 5–6 weeks old male athymic Ncr-nu mice. The tumour volumes in each mouse were determined weekly using calipers and allowed to reach 300 mm$^3$ before treatment. Tumour volumes were calculated using the following formula: tumour volume = $0.5236 \times r_1^2 \times r_2$ ($r_1 < r_2$), where $r_1$ and $r_2$ are radius measurements from tumours. The animals were randomized into treatment groups of 6–8 and administered vehicle (control), flutamide and L-39. The compounds (10 mg ml$^{-1}$) were dissolved in 40% aqueous β-cyclodextrin and administered subcutaneously, on the rear dorsal area of the animal, at a dose level of 50 mg kg$^{-1}$ (~100 µl of drug formulation) daily for 28 consecutive days. A group of mice (6–8) was castrated and injected with the vehicle alone for 28 days. The animals were weighed and tumour volumes measured weekly. The mice were sacrified at the end of the treatment period (1–2 h after the last administered dose) and tumours harvested. The tumours were cleaned, weighed and stored at –70°C until analysis. Blood samples were also collected, centrifuged to obtain serum and stored at –70°C until required.

### Testosterone RIA

Serum and tumour tissues obtained from individual male mice were thawed and homogenized in phosphate buffer (pH 7.4, 0.1 M). The homogenates were centrifuged at 2000 ***g*** for 20 min. Serum (50 µl) and aliquots (50 µl) of the tissue supernatant were used to determine T concentration as described in the $^{125}$I-T assay kit supplied by DSL Inc. Radioactivity was measured using a Packard Cobra II gamma counter.

### DHT RIA

Serum and tumour tissues obtained from individual male mice were thawed and homogenized in phosphate buffer (pH 7.4, 0.1 M). Serum (0.4 ml) and aliquots (0.4 ml) of the tumour supernatant were extracted with 4 ml of hexane:ethanol (98:2) mixture. The extracts were dried under a gentle stream of air, dissolved in sample diluent and used for the determination of DHT concentrations as described in the $^{125}$I-DHT assay kit. Radioactivity was measured using a Packard Cobra II gamma counter.

### PSA ELISA

Serum and tumour tissues obtained from individual mice were thawed and homogenized in phosphate buffer (pH 7.4, 0.1 M). The homogenates were centrifuged at 2000 ***g*** for 20 min. Serum and

© 2000 Cancer Research Campaign

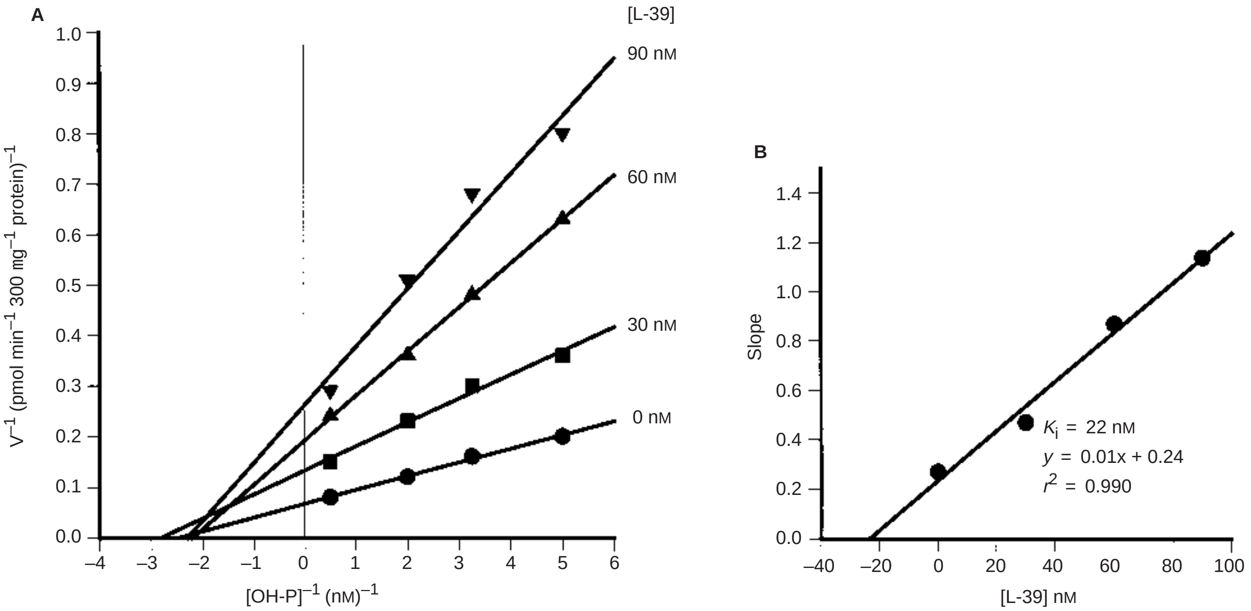

**Figure 2** Inhibition of human testicular microsomal 17α–hydroxylase/$C_{17,20}$-lyase by L-39. (**A**) Lineweaver–Burk plot of enzyme activities at various substrate and inhibitor concentrations, (**B**) slopes of each reciprocal plot against L-39 concentration. Human testicular microsomes were prepared and 17α-hydroxylase/$C_{17,20}$-lyase activity determined as described in Materials and Methods. The $K_m$ and $V_{max}$ values for 17α-hydroxylase/$C_{17,20}$-lyase, under the optimum incubation conditions were 0.48 μM and 40 pmole mg$^{-1}$ protein min$^{-1}$. Each point is the mean of duplicate determination of at least three separate experiments. OH-P = 17α-hydroxypregnenolone. The standard deviations (not shown) were 5–8% of mean values. r ≥ 0.995

aliquots of the tissue supernatant were diluted 1:10 in phosphate buffer (pH 7.4, 0.1 M) and 25 μl used for the determination of PSA concentrations as described in the PSA enzyme-linked immunosorbent assay (ELISA) kit supplied by DSL Inc. The absorbance was measured at 450 nm using a Dynatech MRX plate reader.

### Pharmacokinetic studies

Male Balb/c mice (8–10 weeks old) obtained from NCI (Frederick, MD, USA) were maintained in a controlled environment of about 25°C, 50% relative humidity and 12 h of light and 12 h of dark cycles and allowed free access to food and water. L-39 and flutamide were formulated in 40% β-cyclodextrin in water and a single subcutaneous or intravenous bolus dose was given to mice. The animals were sacrificed at various times up to 24 h after drug administration and blood was obtained by cardiac puncture under light fluothane (Ayerst, New York, NY, USA) anaesthesia.

### HPLC analysis

The high-performance liquid chromatographic (HPLC) system consisted of a Waters® solvent delivery system, Waters 600 controller (Milford, MA, USA) coupled to Waters® 717$^{plus}$ autosampler and a Water® 996 photodiode array detector operated at 254 nm. Chromatographic separation and quantitation of L-39 and the internal standard, L-38, was achieved by reversed phase HPLC on a Water® Novapak® C18 column (3.9 × 150 mm) protected by a Waters® guard cartridge packed with pellicular C18. The mobile phase composition was water/methanol/acetonitrile/acetic acid (25:50:25:0.0001, v/v) and was pumped at a flow rate of 1.0 ml min$^{-1}$. The HPLC analysis was performed at ambient temperature and data acquisition and management was achieved with a Waters® millennium chromatography manager.

### Sample preparation

L-39 and its $\Delta^5$ hydroxy derivative, L-38, were made up to 1 mg ml$^{-1}$ in ethanol and stored in the fridge (4°C) until required. From these stock solutions, dilutions of 1, 10 and 100 μg ml$^{-1}$ in ethanol were prepared for use in construction of calibration curves. For sample preparation, test-tubes containing plasma (250 μl), L-39 and the internal standard, L-38 (10 μg ml$^{-1}$, 6.25 μl), were extracted with diethyl ether (2 × 2 ml) using a vortex mixer for 1 min and centrifugation at 1500 ***g*** for 5 min. The organic layers were evaporated to dryness under a gentle stream of air. The extracts were reconstituted in acetonitrile (250 μl) and loaded into a solid phase Sep-Pak 1cc C18 cartridge (Waters, Milford, MA, USA) pre-washed with methanol (1 ml) for further purification. The cartridge was then eluted with acetonitrile (250 μl), the eluate evaporated to dryness and the residue reconstituted in mobile phase (50 μl) and filtered using 0.2 μm teflon filters for HPLC analysis.

### Calibration curves and HPLC assay validation

The calibration curves for L-39 were constructed by spiking varying amounts of L-39 (0–1 or 0–10 μg ml$^{-1}$ ranges) and the internal standard, L-38 (6.25 μl of 10 μg ml$^{-1}$ or 6.25 μl of 100 μg ml$^{-1}$ respectively) into extraction tubes containing blood (0.25 ml) from untreated animals. The calibration samples were taken through the extraction procedure as described above. An aliquot of the reconstituted extract (10 μl) was injected onto the HPLC column and the ratio of the peak area for L-39 to that of the internal standard (L-38) were plotted against concentrations of L-39. The analytical procedure was validated by determining the

© 2000 Cancer Research Campaign

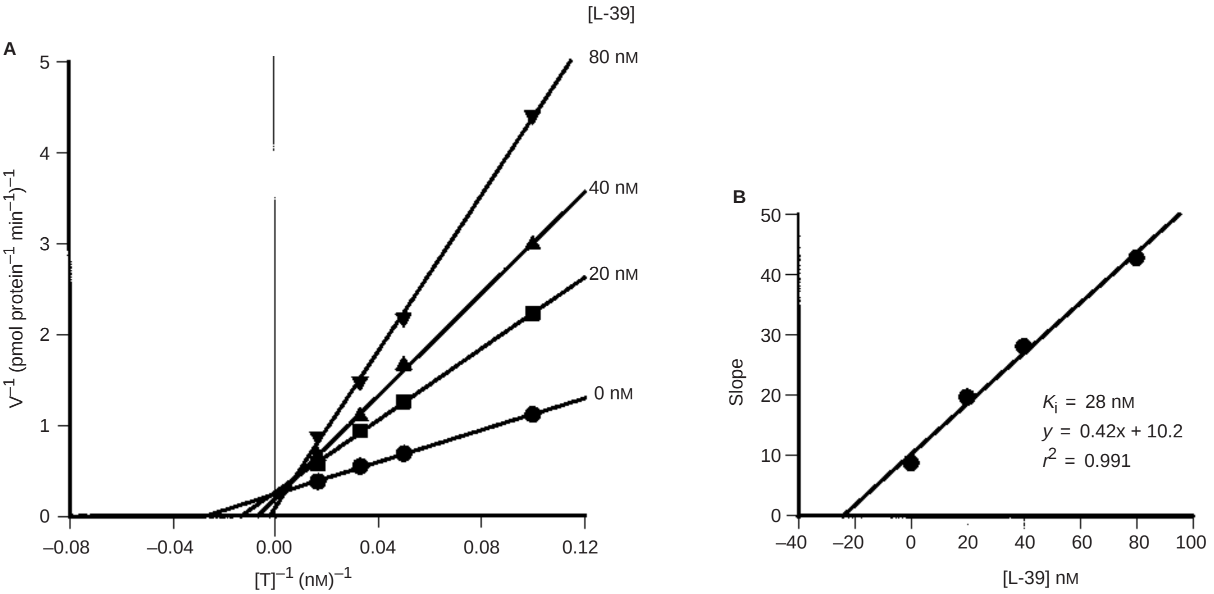

**Figure 3** Inhibition of human prostatic microsomal 5α-reductase by L-39. (**A**) Lineweaver–Burk plot of enzyme activity at various substrate and inhibitor concentrations, (**B**) Slopes of each reciprocal plot against L-39 concentration. Human prostatic microsomes were prepared and 5α-reductase activity determined as described in Materials and Methods. The $K_m$ and $V_{max}$ values for 5α-reductase, under the optimum incubation conditions were 40 nM and 2 pmole mg$^{-1}$ protein min$^{-1}$. Each point is the mean of duplicate determination of at least three separate experiments. The standard deviations (not shown) were 3–7% of mean values. r ≥ 0.995

precision and accuracy of the method. The precision and accuracy of the assay was determined by spiking known concentrations of L-39 into sample tubes containing control plasma and taken through the extraction procedure. The study was repeated on three separate occasions and the coefficient of variation (CV), a measure of precision, and the mean percentage difference, MD(%), a measure of accuracy, were calculated.

### Statistical analysis

Non-compartmental pharmacokinetic calculations were performed using WinNonlin (Scientific consulting Inc.) One-way analysis of variance (ANOVA) on SigmaStat for Windows version 1.0 was used to compare different treatment groups at the 95% confidence level. The Bonferroni post-hoc test was used for determination of significance. A *P*-value of less than 0.05 was considered as statistically significant.

### RESULTS

The $K_m$ and $V_{max}$ values for 17α-hydroxylase/$C_{17,20}$-lyase were 480 nM and 40 pmole mg$^{-1}$ protein min$^{-1}$ respectively, and the reaction was linear with time for up to 1 h of incubation. L-39 showed potent and non-competitive inhibition of human testicular microsomal 17α-hydroxylase/$C_{17,20}$-lyase with an $IC_{50}$ value of 59 nM and $K_i$ of 22 nM (Figure 2). In comparison, ketoconazole, a known competitive inhibitor of 17α-hydroxylase/$C_{17,20}$-lyase, had an $IC_{50}$ value of 78 nM and $K_i$ of 38 nM. L-39 also showed potent and competitive inhibition of 5α-reductase in human prostatic microsomes with $IC_{50}$ and $K_i$ values of 33 and 28 nM respectively (Figure 3). In comparison, finasteride, a clinically available inhibitor of 5α-reductase had an $IC_{50}$ value of 33 nM and $K_i$ of 36 nM. The $K_m$ and $V_{max}$ values for 5α-reductase were 40 nM and 2 pmole mg$^{-1}$ protein min$^{-1}$ respectively, and the reaction was linear with time for up to 20 min of incubation.

Administration of L-39 (50 mg kg$^{-1}$ daily, subcutaneously, for 28 days) to male athymic mice bearing PC-82 tumours significantly reduced tumour volumes and weights compared to controls (Figure 4). The volume of PC-82 prostate cancer xenografts in athymic male nude mice increased by twofold over 28 days in the control group. In comparison, the percentage increase in tumour volume in mice treated with L-39 over 28 days was 1.25-fold. L-39 also significantly diminished PC-82 tumour weights by 70% (Figure 4). The potency of L-39 was similar to that of flutamide in reducing the growth of human PC82 prostate tumour xenografts in nude mice. Gross examination of vital organs such as the liver, kidney and heart and the adrenal gland did not reveal toxicity. Furthermore, the weight of these organs and the body weight of the animals did not change significantly following treatment with L-39 (50 mg kg$^{-1}$). Data from other studies in our laboratory show that maximum suppression of serum testosterone and DHT is achieved by the 50 mg kg$^{-1}$ dose of L-39. Hence, the 50 mg kg$^{-1}$ dose was used in the tumour studies since increasing the dose further did not result in a proportional decrease in serum androgen levels. Furthermore, the 50 mg kg$^{-1}$ dose was used in the tumour studies because no difference in the plasma concentrations of L-39 was seen between the 50 and 100 mg kg$^{-1}$ dose in the pharmacokinetic studies (Figure 5).

L-39 significantly ($P < 0.05$) reduced T and DHT concentrations in PC-82 tumour xenografts by 64% and 94% respectively, although it failed to alter serum androgen levels to any significant extent. L-39 also reduced ($P < 0.01$) serum and tumour PSA levels by 80% and 50% respectively, and to about the same extent as castration and flutamide treatment in mice bearing human prostate cancer xenografts (Figure 6).

On reversed phase HPLC, L-39 was well resolved from the internal standard (L-38) and other endogenous compounds in mouse plasma (Figure 7). The calibration curves derived for L-39

 © 2000 Cancer Research Campaign

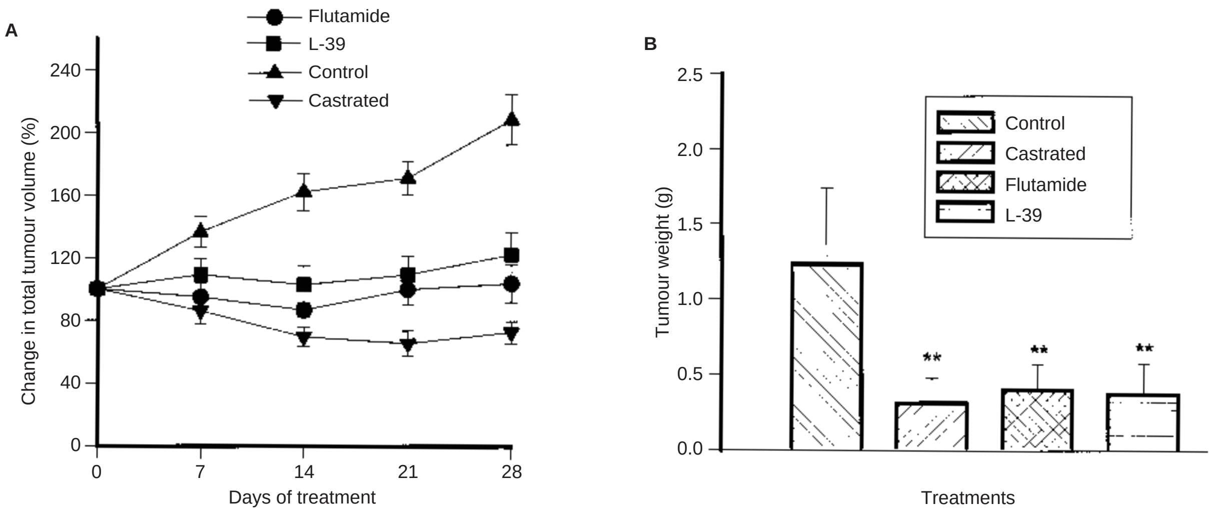

**Figure 4** The effect of L-39 and other endocrine therapies on the growth of human prostate cancer xenografts in male athymic nude mice. (**A**) Tumour volumes, (**B**) tumour weights. Male athymic mice (~20 g) were treated with the compounds listed (50 mg $kg^{-1}$ $day^{-1}$, s.c., for 28 consecutive days). Tumour volumes were measured weekly. The animals were sacrificed after 28 days and the tumours harvested and weighed. Values are the means ± standard error from 6–8 mice. **P* < 0.05, and ** *P* < 0.01, compared to the control group

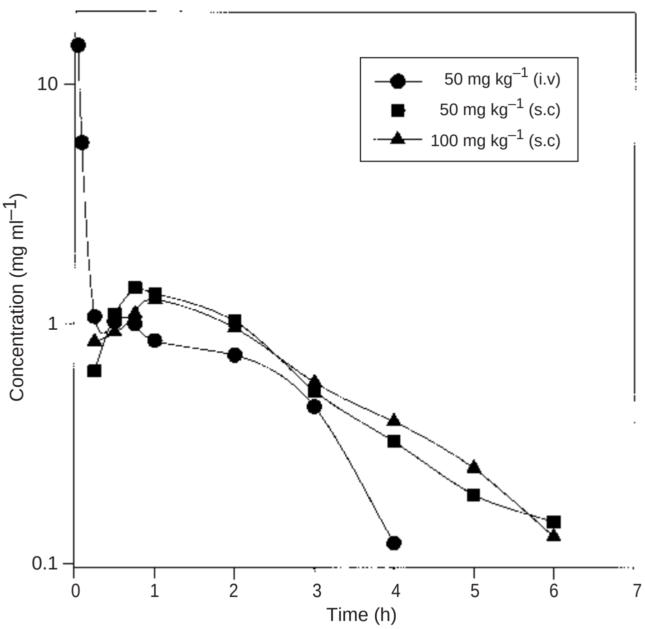

**Figure 5** Pharmacokinetics profile of L-39 following administration of a single subcutaneous or intravenous bolus dose to male mice. Male Balb/c mice (~20 g) were injected with L-39 (50 and 100 mg $kg^{-1}$) as a single bolus dose and blood collected at predetermined time points. The samples were analysed as described in Materials and Methods. Each data point represents the mean of plasma concentrations obtained from at least three mice. The standard deviations (not shown) were ± 3–7% of the mean values

**Table 1** Pharmacokinetic parameters of L-39 in mice following a single intravenous or subcutaneous bolus dose

| Parameter | Subcutaneous | | Intravenous |
|---|---|---|---|
| | 50 mg $kg^{-1}$ | 100 mg $kg^{-1}$ | 50 mg $kg^{-1}$ |
| $K_{el}$ ($h^{-1}$) | 0.46 ± 0.02 | 0.53 ± 0.03 | 1.05 ± 0.05 |
| $T_{1/2>}$ (h) | 1.51 ± 0.08 | 1.31 ± 0.06 | 0.66 ± 0.04 |
| $AUC_{0-\infty}$μg $ml^{-1}.h^{-1}$) | 4.09 ± 0.16 | 4.04 ± 0.20 | 14.41 ± 0.56 |
| Cl (l $h^{-1}$ $kg^{-1}$) | 12.24 ± 0.48 | 24.78 ± 1.0 | 3.47 ± 0.14 |
| $V_d$ (l $kg^{-1}$) | 26.73 ± 1.04 | 46.80 ± 1.84 | 3.30 ± 0.13 |
| MTR (h) | 2.52 ± 0.11 | 2.41 ± 0.10 | 1.15 ± 0.05 |
| $T_{max}$ (h) | 0.75 ± 0.0 | 1.0 ± 0.0 | – |
| $C_{max}$ (μg $ml^{-1}$) | 1.42 ± 0.05 | 1.30 ± 0.04 | – |

Male mice (25 ± 5 g) were injected with L-39 (50 or 100 mg $kg^{-1}$, s.c. or

50 mg $kg^{-1}$ i.v). The animals were sacrificed and blood collected at predetermined time points. plasma concentrations of L-39 were determined by HPLC, as described in Materials and Methods. Values represent the means ± standard deviations from at least three mice.

were linear and reproducible (data not shown) and the inter- and intra-assay variability was less than 10%. The limit of detection for L-39 in mouse plasma was 0.05 μg $ml^{-1}$. The HPLC assay was validated and used to monitor L-39 concentrations in mice plasma. The typical mean plasma concentration–time profiles of L-39 after administration of a single bolus dose of 50 mg $kg^{-1}$, intravenously or subcutaneously, to male mice are shown in Figure 5. Following intravenous administration, the plasma concentration of L-39 declined exponentially with a mean half-life of 0.66 h and a terminal elimination rate constant of 1.052 $h^{-1}$. L-39 was rapidly cleared (total clearance of 3.5 l $h^{-1}$ $kg^{-1}$) from the systemic circulation and was not detectable 4 h after administration. The calculated non-compartmental pharmacokinetic parameters based on the plasma concentration profile following intravenous administration of L-39 are shown on Table 1. L-39 was detected in mice plasma from 5 min to 6 h following subcutaneous administration of a 50 mg $kg^{-1}$ dose. The blood levels of L-39 peaked at about 0.75 h and then declined exponentially (Figure 5) with a mean half-life of 1.51 h and a terminal elimination rate constant of 0.46 per h. The bioavailability following subcutaneous administration of L-39 (50 mg $kg^{-1}$) was 28.5%. The calculated non-compartmental pharmacokinetic parameters based on the plasma concentration profile following subcutaneous administration of L-39 are shown on Table 1. The terminal slope of the plasma concentration-time profile of L-39 following subcutaneous administration is shallower compared to the terminal slope following intravenous dosing. Thus, the pharmacokinetic study indicates that

*© 2000 Cancer Research Campaign*

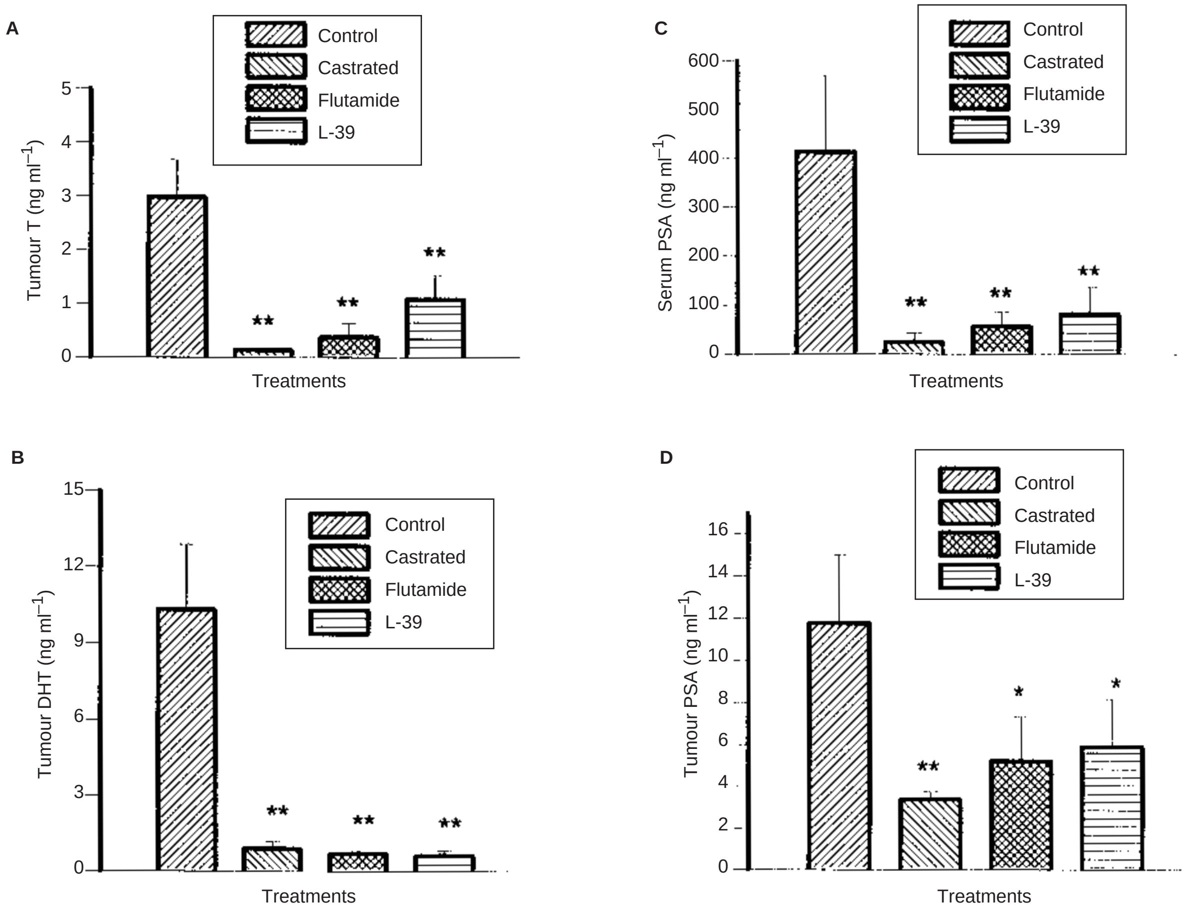

**Figure 6** Effects of L-39 on (**A**) tumour T, (**B**) tumour DHT, (**C**) serum PSA and (**D**) tumour PSA levels in athymic nude mice. Male athymic mice (~20 g) were treated with the compounds listed (50 mg kg$^{-1}$ day$^{-1}$, s.c., for 28 consecutive days). The animals were sacrificed after 28 days and blood was collected and tumours harvested. Serum and tumor concentrations of testosterone (T), dihydrotestosterone (DHT) and PSA were determined by RIA and ELISA as described under Materials and Methods. Values represent the means ± standard error from 6–8 mice.* $P < 0.05$, and ** $P < 0.01$, compared to the control group

L-39 persists in the blood longer when administered subcutaneously. When a higher dose of L-39 (100 mg kg$^{-1}$) was administered subcutaneously to male mice, the plasma concentration versus time curve was almost superimposable on the plasma concentration versus time curve for the 50 mg kg$^{-1}$ following subcutaneous administration of L-39. Hence, the area under the plasma concentration–time profile did not change significantly after subcutaneous administration of the higher dose (Table 1). However, the volume of distribution and the total clearance were increased significantly at the higher dose.

### DISCUSSION

17α-hydroxylase/$C_{17,20}$-lyase catalyses the early step in the biosynthesis of T and other androgens in both the testes and the adrenal glands, while 5α-reductase converts T to DHT in the prostate gland (Saunders, 1963). Both T and its metabolite, DHT, promote prostatic growth and cancer. Thus, inhibition of both enzymes would be expected to result in diminished levels of circulating T and DHT and therefore serve as a useful strategy for developing new treatments for prostate cancer (Nnane et al, 1998, 1999). Several inhibitors of 17α-hydroxylase/$C_{17,20}$-lyase and/or 5α-reductase have been described previously, however, they have a number of limitations. Ketoconazole, currently used in the treatment of prostate cancer, is an inhibitor of several P450 enzymes including 17α-hydroxylase/$C_{17,20}$-lyase (Small et al, 1997), and causes side-effects such as nausea, dry skin, asthenia, etc. (Trachtenberg, 1984). Finasteride, an inhibitor of 5α-reductase, induces accumulation of T (Peters and Sorkin, 1993) and has no activity against 17α-hydroxylase/$C_{17,20}$-lyase (Nnane et al, 1998). L-39 is a dual inhibitor of 17α-hydroxylase/$C_{17,20}$-lyase and 5α-reductase. L-39 showed non-competitive inhibition and probably binds strongly to the apoprotein of 17α-hydroxylase/$C_{17,20}$-lyase (Figure 2). L-39 was a potent competitive inhibitor of 5α-reductase. The observed anti-tumour effects of L-39 are therefore due to its potent inhibition of androgen synthesis, at least in part. L-39 (5 μM) has also been shown to manifest anti-androgenic activity in cultures of human prostate cancer cell lines (LNCaP) by preventing the labelled synthetic androgen R1881 (5 nM) from binding to the androgen receptors (Long et al, 1999). The interaction of L-39 with the androgen receptor together with its potent inhibition of androgen synthesis makes L-39 a promising lead compound for the treatment of prostate cancer.

Furthermore, L-39 was effective in vivo in reducing tumour volumes and weights and PSA and androgen levels in tumour tissues. These effects were produced without a significant change

*© 2000 Cancer Research Campaign*

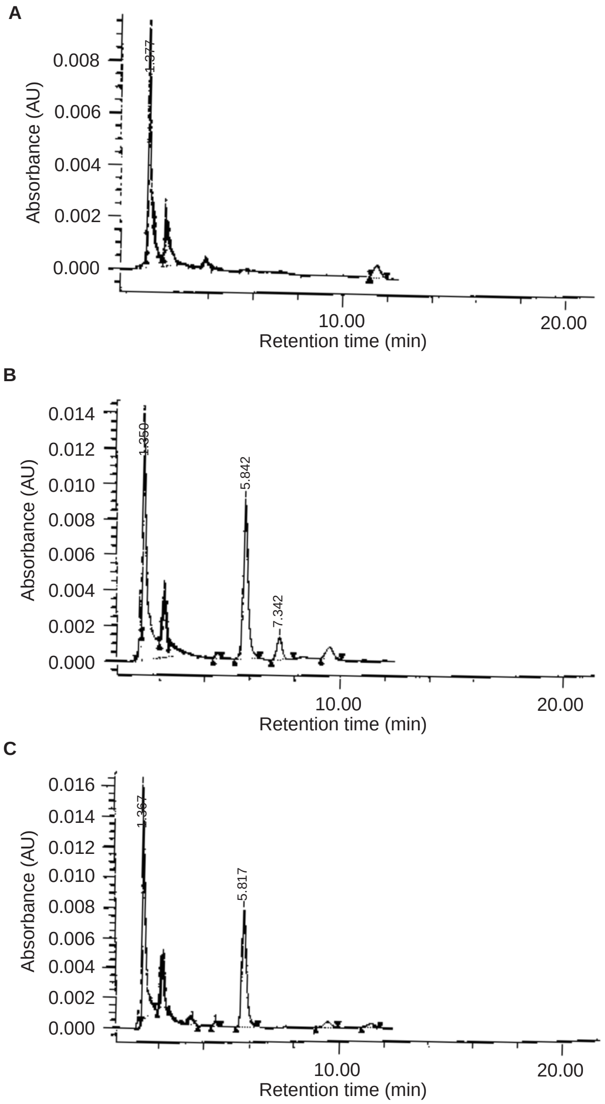

**Figure 7** Typical HPLC chromatogram of L-39 and L-38 (internal standard) extrated from mouse plasma. (**A**) untreated mouse plasma, (**B**) untreated mouse plasma spiked with L-39 and L-38, (**C**) plasma sample from mouse treated with L-39. The retention times for L-39 and the internal standard L-38 were 5.8 and 7.3 min respectively the weights of vital organs and the body weight of the animals following treatment with L-39 (50 mg $kg^{-1}$), an indication that the compound is well tolerated by the animals.

Surgical castration is the traditional approach to lower androgen levels in vivo and was used in this investigation as the standard for comparison. This novel steroid was as effective as castration and flutamide in reducing androgen concentration and PSA levels in tumour tissues. Flutamide, an anti-androgen, is currently used clinically in combination with LHRH analogue, leuprolide (Crawford et al, 1989). However, the duration of its effect is often limited by the development of androgen receptor mutants that respond to flutamide as an agonist. Tumour growth and weight are regulated by both T and DHT, albeit to a greater extent by the latter (Wilson, 1996). Thus, the significant reduction in the levels of androgens by L-39 may explain the corresponding reduction in tumour volume and weight observed after treatment of nude mice bearing PC-82 tumors with L-39. The serum marker, PSA, has been used to determine the status of prostate cancer in patients and is a valid end point for assessing progression of the disease (Roach, 1996). Our novel androstene derivative, L-39, was effective in lowering PSA levels ($P < 0.001$) in mice bearing human PC-82 tumours and its effectiveness was similar to that of castration ($P > 0.05$). Other studies in our laboratory indicate that L-39 inhibits proliferation LNCaP human prostate cancer cells in culture and is effective at slowing LNCaP human prostatic cells grown in male SCID mice as tumours (Long et al, 1999). This finding suggests that L-39 may also be effective in treating tumours with the mutated androgen receptor.

Our results indicate that although L-39 is a potent inhibitor of androgen synthesis in vitro and is effective in reducing serum and tumour androgen concentrations, its effectiveness in vivo on T levels is weaker than that of castration. The half-life of L-39 was about 1.51 h after subcutaneous administration. The relatively short half-life of the compound may explain its efficacy in vivo since it was less than expected based on its in vitro potency. It may be necessary to administer L-39 more frequently or increase its bioavailability in order to increase efficacy in vivo. Although the in vivo activity of L-39 was less than expected, the observed anti-tumour effect suggests that the initial concentrations of L-39 in circulation may be sufficiently high for the compound to irreversibly bind and saturate the 17α-hydroxylase/$C_{17,20}$-lyase. In fact, other steroidal compounds with a 16–17 double bond have been shown to be irreversible inhibitors of 17α-hydroxylase/$C_{17,20}$-lyase (Jarman et al, 1990). The pharmacokinetics study indicate that L-39 persists in the blood slightly longer when administered subcutaneously. This persistence may be due to slower release of L-39 into the systemic circulation from the subcutaneous depot and may be a more useful method of administration for achieving levels that inhibit the enzyme and bind androgen receptors in the tumour. The relatively low bioavailability (28.5%) of the compound may also be limiting its efficacy in vivo. The area under the curve did not increase proportionately with an increase in the dose of L-39 following subcutaneous administration. It would appear, therefore, that L-39 exhibits non-linear pharmacokinetics. This observation suggests that the processes of uptake, distribution and metabolism of L-39 are saturable and that the doses used are sufficiently high to cause saturation of these processes. Initial observations in our laboratory indicate that L-39 is metabolized to a more polar metabolite in mice although the metabolite has not yet been characterized. Moreover, L-39 is poorly soluble in water and was formulated in 40% aqueous β-cyclodextrin. Although a homogeneous solution was obtained with 40% aqueous β-cyclodextrin, L-39 may precipitate at the site of injection. This may affect the rate and extent of L-39 that is systemically available and that may explain, at least in part, the observed non-linear kinetics of L-39. The relatively large volume of distribution suggests that L-39 may be extensively bound to plasma proteins or is distributed extensively within the extravascular tissues. Studies on the uptake and distribution of the compound into the testis and tumours and on protein binding are in progress. Studies are also in progress to improve the formulation of L-39 such that adequate levels of the drug are maintained in the systemic circulation for a longer period of time in order to increase it efficacy in vivo.

In conclusion, the findings in this investigation indicate that L-39 is a potent inhibitor of androgen synthesis and is also effective in reducing the growth of human PC-82 tumours in nude mice. Recent studies in our laboratory have also shown that L-39 manifests anti-androgenic activity in vitro and that it blocked the

© 2000 Cancer Research Campaign mutated androgen receptor in LNCaP cells. Thus, the compound was more effective than flutamide in mice with LNCaP tumours (Long et al, 1999). Although inhibition of androgen synthesis appear to be the primary mode of action of L-39, the overall anti-tumour effect, due to its other mechanisms of action, may be of benefit in treating prostate cancer. In fact, the ability of L-39 to interact with the androgen receptor may also contribute significantly to the observed anti-tumour activity in vivo. Although improvements in the formulation and pharmacokinetic profile are necessary, L-39 is a promising lead compound for the treatment of hormone-dependent cancer of the prostate.

### ACKNOWLEDGEMENTS

This work was supported by NIH grant No. CA-27440 and funds from Paramont capital Inc.

### REFERENCES

Angelastro MR, Laughlin ME, Schatzman GL, Bey P and Blohm TR (1989) 17α-(cyclopropylamino)-androsta-5-en-3α-ol, a selective mechanism based inhibitor of cytochrome P-450$_{17\alpha}$ (steroid 17α-hydroxylase/$C_{17,20}$lyase). *Biochem Biophys Res Commun* **162**: 1571–1577

Ayub M and Levell MJ (1987) Inhibition of testicular 17α-hydroxylase and 17,20-lyase but not 3-hydroxysteroid dehydrogenase–isomerase or 17-hydroxysteroid oxidoreductase by ketoconazole and other imidazole drugs. *J Steroid Biochem* **28**: 521–531

Bruchovsky N and Wilson JD (1968) The conversion of testosterone to 5α-androsta-17α-ol-3-one by rat prostate in vivo and in vitro. *J Biol Chem* **243**: 2012–2021

Crawford ED, Eisenberger MA, McLeod DG, Spaulding JT, Benson R, Dorr FA, Blumenstein BA, Davies MA and Goodman PJ (1989) A controlled trial of leuprolide with and without flutamide in prostatic carcinoma. *N Engl J Med* **321**: 419–424

Cunningham GR and Hirshkowitz M (1995) Inhibition of steroid 5α-reductase with finasteride: sleep-related erections, potency and libido in healthy men. *J Clin Endocrinol Metab* **80**: 1934–1940

Dijkman GA and Debruyne FM (1996) Epidemiology of prostate cancer. *Eur Urol* **30**: 281–295

Grigoryev DN, Long BJ, Nnane IP, Njar VCO, Liu Y and Brodie AMH (1999) Effects of new 17α-hydroxylase/$C_{17,20}$-lyase inhibitors on LNCaP prostate cancer cell growth in vitro and in vivo. *Br J Cancer*. **81**: 622–630

Jarman M, Barrie SE, Deadman JJ, Houghton J and McCague R (1990) Novel inhibitors of enzymes of androgen biosynthesis. *J Med Chem* **33**: 3050–3055

Klus GT, Nakamura J, Li J, Ling Y, Son C, Kemppainen JA, Wilson EM and Brodie AMH (1996) Growth inhibition of human prostate cells in vitro by novel inhibitors of androgen synthesis. *Cancer Res* **56**: 4956–4964

Labrie F, Dupont A, Belanger A, Lacoursiere Y, Raynaud JP, Husson JM, Gareau J, Fazekas ATA, Sandow J, Monfette G, Girard JG, Emond J and Houle JG (1983) New approach in the treatment of prostate cancer: complete instead of partial withdrawal of androgens. *Prostate* **4**: 579–594

Li J, Li Y, Son C, Banks P and Brodie A (1992) 4-Pregnen-3-one-20α-carboxyaldehyde: a potent inhibitor of 17α-hydroxylase/$C_{17,20}$lyase and of 5α-reductase. *J Steroid Biochem Mol Biol*, **42**: 313–321

Li J, Li Y, Son C and Brodie AMH (1995) Inhibition of androgen synthesis by 22-hydroximino-23, 24-bisnor-4-cholen-3-one. *Prostate* **26**: 140–150

Li JS, Li Y, Son C and Brodie A (1996) Synthesis and evaluation of pregnane derivatives as inhibitors of human testicular 17α-hydroxylase/$C_{17,20}$lyase. *J Med Chem* **39**: 4335–4339

Ling Y, Li J, Liu Y, Kato K, Klus GT and Brodie A (1997) 17-Imidazolyl, pyrazolyl and isoxazolyl androsteine derivatives. Novel steroidal inhibitors of human cytochrome $C_{17,20}$-lyase (P-450$_{17\alpha}$). *J Med Chem* **40**: 3297–3304

Long BJ, Grigoryev DN, Nnane IP, Liu Y, Ling Y, Wang X and Brodie A (1999) In vitro and in vivo inhibition of LNCaP prostate cancer cell growth by novel inhibitors of androgen synthesis. In: *Proceedings of the 90th Annual Meeting of American Association for Cancer Research*, Philadelphia, 1999, vol. 40, p 64 (abstract).

Lowry OH, Roseborough NS, Farr AL and Randall RS (1951) Protein measurement with the folin phenol reagent. *J Biol Chem* **193**: 265–275

Nakajin S, Takahashi K and Shinoda M (1989) Inhibitory effects and interaction of stanozolol with pig testicular cytochrome P-450 (17α-hydroxylase/$C_{17,20}$lyase). *Chem Pharm Bull* **7**: 1855–1858

Njar VCO and Brodie AMH (1999) Inhibitor of 17α-hydroxylase/$C_{17,20}$lyase (CYP17): potential agents for the treatment of prostate cancer. *Current Pharm Design* **5**: 163–180

Njar VCO, Kato K, Nnane IP, Grigoryev DN, Long BJ and Brodie AMH (1988) Novel azolyl steroids; potent inhibitors of human cytochrome 17α-hydroxylase/$C_{17,20}$-lyase (P-450$_{17\alpha}$): potent inhibitors for the treatment of prostate cancer. *J Med Chem* **41**: 902–912

Nnane IP, Kato K, Liu Y, Lu Q, Wang X, Ling Y and Brodie A (1998) Effects of some novel inhibitors of $C_{17,20}$-lyase and 5α-reductase in vitro and in vivo and their potential role in the treatment of prostate cancer. *Cancer Res* **58**: 3826–3832

Nnane IP, Kato K, Liu Y, Long BJ, Lu Q, Wang X, Ling Y and Brodie A (1999) Inhibition of androgen synthesis in human testicular and prostatic microsomes and in male rats by novel steroidal compounds. *Endocrinology* **140**: 2891–2897

Peters DH and Sorkin M (1993) Finasteride: a review of its potential in the treatment of benign prostatic hyperplasia. *Drugs* **46**: 177–208

Roach M (1996) The role of PSA in the radiotherapy of prostate cancer. *Oncology* **10**: 1143–1153

Rosenberg AG and von Eschenbach AC (1990) Hormonal therapy for prostate cancer. *Semin Surg Oncol* **6**: 71–76

Saunders FJ (1963) Some aspects of relation of structure of steroids to their prostate stimulating effects. In: *Prostate and Related Tissues* (National Cancer Institute monograph No 12), Vollmer EP (ed), pp. 139–159. US Government Printing Office: Washington, DC

Small EJ, Baron AD, Fippin L and Apodaca D (1997) Ketoconazole retains activity in advanced prostate cancer patients with progression despite flutamide withdrawal. *J Urol* **157**: 204–1207

Trachtenberg J (1984) Ketoconazole therapy in advanced prostatic cancer. *J Urol* **132**: 61–63

Wilson JD (1996) Role of dihydrotestosterone in androgen action. *Prostate* **6**: 88–92

 © 2000 Cancer Research Campaign
